# Network analysis based on big data in social media of Korean adolescents’ diet behaviors

**DOI:** 10.1371/journal.pone.0273570

**Published:** 2022-08-25

**Authors:** JongHwi Song, SooYeun Yoo, JunRyul Yang, SangKyun Yun, YunHee Shin

**Affiliations:** 1 Division of Software, Yonsei University, Wonju, Gangwon-do, Republic of Korea; 2 Department of Nursing, Wonju College of Medicine, Yonsei University, Wonju, Gangwon-do, Republic of Korea; UAB School of Medicine, UNITED STATES

## Abstract

Adolescents are increasingly interested in weight control; hence, proper health education is important for helping them control their weight properly. This study was designed to pick out social media words that express adolescents’ diet behaviors, and identify the associations and types between such words and the behaviors. It used text-mining techniques and semantic network analysis for related big data collected from the Internet on adolescents’ diet behaviors. Text mining was used to extract meaningful information from unstructured text data, whereas semantic network analysis was used to understand the relationships between keywords. The top five keywords were “obesity,” “health,” “exercise,” “eat,” and “increase” in online news, and “exercise,” “eat,” “weight loss,” “obesity,” and “health” in blogs. The betweenness centrality of “appearance” was particularly higher than that of other centralities in online news. As a result of the CONCOR analysis, eight clusters each were identified in online news and blogs. This study’s results will serve as a basis for weight management-related intervention strategies, reflecting the perspectives of adolescents. It also has significance as basic data to provide correct information, and establish desirable weight control in the future.

## Introduction

As adolescents are increasingly interested in weight control and diet behaviors, proper health education is important to help them in controlling their weight properly. The obesity rate among Korean adolescents (12.1%) is increasing yearly, with 34.6% adolescents attempting to lose weight and 23.9% having a distorted body image [1]. Likewise, the National Health and Nutrition Examination Survey data revealed that during 2007–2008, approximately 18.1% of 12–19 year-old in the United States were obese, which increased to 21.2% during 2017–2018 [2]. Maintaining healthy diet behaviors is a challenge for adolescents. In recent years, diet education interventions have increasingly relied on computing and information technologies, especially mobile platforms and social media [3]. As adolescents display a high level of smartphone and social media usage, they are more likely to use these platforms for monitoring their health [4]. Korean adolescents’ Internet usage time, excluding for learning purposes, was 112.2 and 189.6 min on weekdays and weekends, respectively [1]. Most adolescents already rely on smartphones to search for health information [4].

While Internet use for education and communications has potential advantages, there are growing concerns about problematic Internet use. As such, providing correct information is important considering the high rate of weight loss attempts among adolescents, and the large amount of time that they spend on smartphones. Additionally, a survey on adolescents’ health education needs found that they strongly desired advice on weight control [5]. A meta-analysis of studies on improving adolescents’ health habits revealed that greater beneficial effects on health behaviors can be guaranteed by providing adolescents with helpful information to motivate them [6].

Big data are not merely a voluminous quantity of data that can be collected, stored, and analyzed [7], or the technology for processing large amounts of data [8]; rather, their essence lies in the value than can be created from such data. The core of big data technology lies in its ability to provide valuable new information and services by analyzing information that pours in. Therefore, collecting and analyzing online information on diet behaviors—a topic that adolescents are most interested in—will provide useful basic information to adolescents, who spend long hours on social media, and help them to grow into healthy adults.

Network analysis is a useful method for deriving the characteristics of the network type, and explaining the features of topics of interest by relationship [9]. It can be used for analyzing users’ thought patterns based on content posted on social media, using text-mining techniques, and is, therefore, useful for understanding the context of connections between networked content [10]. Furthermore, such analyses and visualizations have the advantage of facilitating a grasp of the knowledge structure of the phenomenon of interest, and showing the direction [11]. Therefore, by analyzing and categorizing the connectivity of big data-based collection, analysis, and processing, the characteristics and structure of the contents related to the diet behaviors of adolescents—a phenomenon of interest—are identified.

Previous studies that attempted big data-based network analysis on adolescents had considered their peer relationships, smoking and drinking experiences [12], and peer networks according to their physical factors [9], and used semantic network analysis for assessing the knowledge structure of students with severe and multiple disabilities [13]. Another study on physical activity and exercise in school-aged youth aimed to provide a solution by analyzing a large number of scientific articles using text mining [14]. A study has also been conducted to analyze Korean adolescents’ perceptions of sports and physical activities through big data analysis over the last 10 years, and provide research data and statistical direction with regard to their participation in such activities [15]. Under the premise that social media plays an important role in young people’s daily lives, a study describing a big data approach to social media has been presented. The study exemplified this approach by analyzing an ad hoc dataset from the pro-eating disorder forum of a social media website [16]. During a review of previous studies, it was difficult to find a study that had used network analysis based on big data in social media to explore the diet behaviors of adolescents, despite the increasing number of studies using big data-based network analysis in various academic fields [17].

Therefore, this study was designed to provide basic data for establishing strategies to prevent adolescent obesity, which is increasing yearly, and establish desirable weight control, using social media for big data-based network analysis of Korean adolescents’ diet behaviors. Hence, its purpose was to identify social media words that expressed adolescents’ diet behaviors, and identify the associations between such words and their types.

## Materials and methods

**Fig 1:**
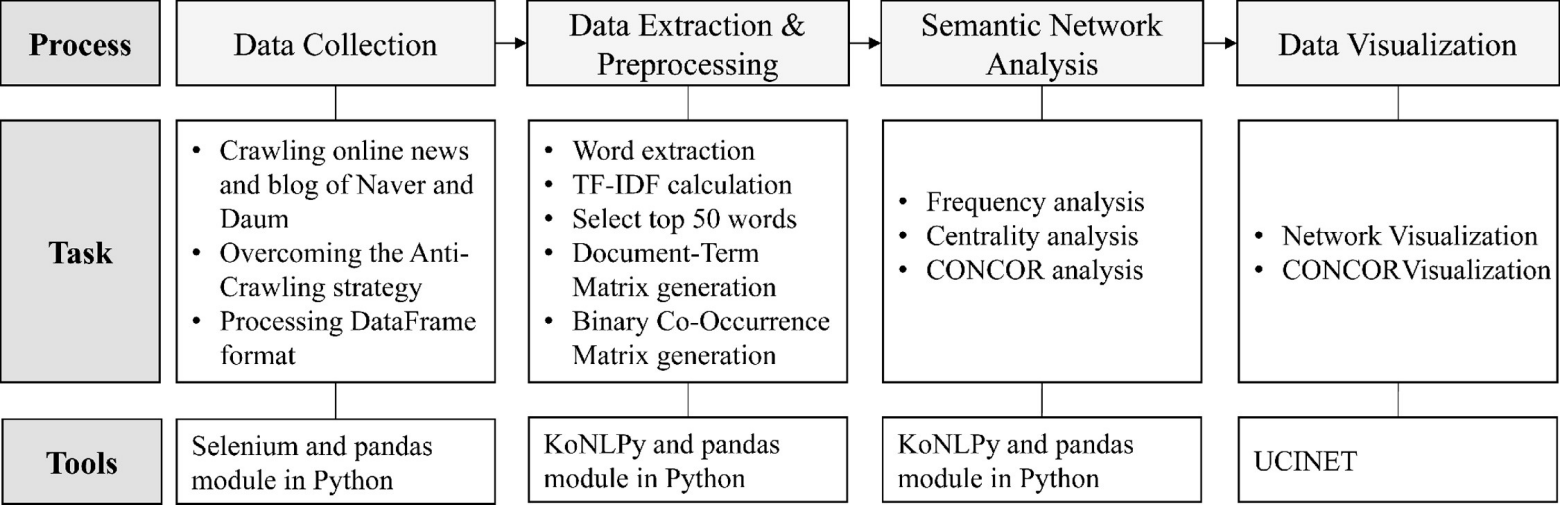
Data collection and analysis process for adolescents’ diets.

The diet behaviors of adolescents were analyzed using text-mining techniques and semantic network analysis for related big data collected from the Internet. Text mining is the process of extracting meaningful information from unstructured text data to explore key topics and trends from multiple perspectives. Semantic network analysis is used to understand the relationships between keywords. In this study, the following analysis process was established to understand the meaning of words based on their associations related to adolescents’ diets in online news articles and blogs. The overall analysis process is shown in Fig 1.

### Data collection

We collected data on adolescents’ diet from online news and blogs in Naver [18] and Daum [19], which are the two largest portals in Korea. Using the search keyword “adolescents’ diet,” we collected 1,423 online news articles from Naver News and 1,733 blog posts from the Naver and Daum blogs.

Online news article texts were collected only from Naver because almost all Korean news articles can be found in Naver, and articles inevitably got duplicated when both Naver and Daum were searched. Many articles were duplicated in Naver because various news media provide the same articles. Hence, we removed duplicate articles using cosine similarity, which refers to the degree of similarity between vectors measured, using cosine values of the angles between two vectors in space. As Naver and Daum blogs are rarely duplicated, blog posts were collected from both the sites. Naver and Daum represent 74.1 and 18.7% share of Korean blog sites, respectively [20].

The data were collected using a web crawling program implemented in Python. We overcame the anti-crawling strategy of websites using the Selenium library, which automates web browsers. Web-crawling data were processed using the BeautifulSoup Library, and saved in the DataFrame format of the Pandas library.

### Data extraction and preprocessing

Data preprocessing was performed using KoNLPy, an open source Python library for natural language processing in Korea [21]. The collected data were refined using nouns, verbs, and adjectives, except for special characters and symbols, through morphological analysis using KoNLPy. After extracting the word list, Term Frequency-Inverse Document Frequency (TF-IDF) was calculated from a morpheme of one or more words.

TF-IDF is a statistical measure that evaluates how relevant a word is to a document in a collection of documents. It is performed by multiplying two metrics: the term frequency of the document and the inverse document frequency of the word across a set of documents. This weight value is mainly used to obtain similarity in documents, as well as the importance of search results in searches and of specific words within a document.

Not every word in the dataset was considered as the co-occurrence matrix node, but by using the word-frequency lists, words whose frequencies were less than certain cut-off values were excluded. In addition, the words that commonly appeared across all datasets were also ruled out because they are less meaningful in detecting differences in the semantic networks derived from distinct datasets [22, 23].

For keyword selection, it is desirable to select the most appropriate word for the research topic, while referring to the opinions of experts [24]. Therefore, in this study, the top 50 words were selected based on their TF-IDF values, which reflected the opinions of a high school counselor, public health teacher, and network analysis expert. When selecting words, unrelated words, such as “person” and “society,” were excluded, and words similar in meaning were incorporated. For example, all the frequencies of “fat,” “overweight,” and “gain weight,” which were similar to that of “obesity,” were added to the frequency of “obesity.”

Based on these 50 selected words, a Document-Term Matrix (DTM) was generated to represent the frequency of each word appearing in multiple articles and blogs. A DTM is meaningful in that it can quantify the relationship between words and documents. Subsequently, a Co-Occurrence Matrix (COM) was generated to determine the frequency of simultaneous appearances of words in the entire document.

Because the generated COM is complex to analyze, using the median of its all elements as cut-off value, it was transformed into a binary matrix by changing to 1 for a value higher than the median value, and 0 for a value lower than the median value. This task involved creating a loose relationship by simply comparing excessively dense values with 1 and 0 in the network analysis. We used the binary matrix as keyword COM in semantic network analysis. A network represented by keyword COM is an unweighted and undirected network.

### Semantic network analysis and visualization

Semantic network analysis was used to understand the relationship between refined words related to adolescents’ diet. It is a mixed method of social network analysis that identifies the structural characteristics of social phenomena, and uses data mining techniques for analyzing unstructured big data [22]. To intuitively recognize the co-occurrence relationship among the refined words in the social media data, the COM that was created in the previous section was visualized using NetDraw, a network visualization program [25].

To identify the connection structure of words related to adolescents’ diet, NetworkX, a Python package [26], was used to analyze the following network centralities: 1) degree centrality—the number of nodes a particular node is connected to; 2) betweenness centrality—a measure of the mediation role of a node in a network; 3) closeness centrality—the inverse of the mean distance to all other nodes, which indicates how close a node is to all other nodes; and 4) eigenvector centrality—a measure of the influence of a node in a network [27].

A CONvergence analysis of an iterative CORrelation (CONCOR) was performed to identify mutually exclusive subgroups in the semantic network. CONCOR repeatedly partitions nodes into subsets based on structural equivalence, and analyzes Pearson’s correlations to search for groups with certain levels of similarity. It forms clusters, including nodes with similarities to each other [28]. This method is generally used to identify the relationship between simultaneous nodes of keywords across all possible keywords, by finding clusters of similar keywords [29]. We used UCINET 6.0 [30] to perform the CONCOR analysis, and the results were visualized using NetDraw.

## Results

### Keyword frequency related to adolescents’ diet

The word-frequency analysis of online news and blogs (Tables 1 and 2, respectively) resulted in the top 50 words. The top five keywords were: “obesity” and “health,” followed by “exercise,” “eat,” and “increase” in the online news, and “exercise,” “eat,” “weight loss,” “obesity,” and “health” in the blogs.

**Table 1:** Frequencies of 50 keywords related to adolescents’ diets in online news.

Rank	Keyword	Freq	Rank	Keyword	Freq	Rank	Keyword	Freq
1	obesity	3371	18	problem	982	35	milk	618
2	health	3018	19	make	933	36	prevention	609
3	exercise	2942	20	education	882	37	stress	551
4	eat	2592	21	method	867	38	menu	480
5	increase	2384	22	use	866	39	plan	452
6	food	2375	23	product	865	40	protein	451
7	weight loss	1842	24	diverse	864	41	advertisement	450
8	weight	1660	25	activity	819	42	information	424
9	intake	1524	26	needed	815	43	eat nothing	418
10	management	1520	27	investigation	776	44	side effect	399
11	effect	1327	28	life	775	45	consult	383
12	follow	1294	29	habit	758	46	muscle	376
13	appearance	1261	30	meal	726	47	video	366
14	fat	1252	31	calorie	719	48	take dose	363
15	treatment	1225	32	function	669	49	anorexia	315
16	prohibition	1191	33	school	657	50	entertainer	283
17	program	1091	34	vitamin	646			

**Table 2:** Frequencies of 50 keywords related to adolescents’ diets in blogs.

Rank	Keyword	Freq	Rank	Keyword	Freq	Rank	Keyword	Freq
1	exercise	9947	18	follow	1170	35	make	707
2	eat	4937	19	calorie	1128	36	herbal medicine	688
3	weight loss	4712	20	appearance	1105	37	muscle	687
4	obesity	4367	21	worry	1085	38	posture	642
5	health	3318	22	school	1082	39	treatment	641
6	food	2464	23	function	1067	40	stress	636
7	management	2386	24	product	948	41	diverse	619
8	weight	2346	25	program	944	42	problem	583
9	consult	1641	26	ingredient	882	43	life	581
10	video	1581	27	habit	868	44	friend	580
11	Konjac	1573	28	use	864	45	appetite suppressant	552
12	intake	1354	29	needed	840	46	activity	488
13	method	1301	30	take dose	817	47	inquiry	477
14	prescription	1281	31	meal	794	48	correction	475
15	increase	1234	32	side effect	775	49	physical constitution	451
16	fat	1221	33	advertisement	756	50	skip a meal	357
17	effect	1195	34	menu	726			

### Analysis of centralities of keywords related to adolescents’ diets

Table 3 shows the network centralities analyzed using the keyword COM for online news. As the keyword “increase” had the most connections with other keywords, it had the highest degree centrality, followed by “obesity,” “health,” “exercise,” “food,” “eat,” “weight loss,” and “management”; the highest betweenness centrality, followed by “appearance,” “health,” “obesity,” “exercise,” “food,” “follow,” and “management”; the highest closeness centrality, followed by “obesity,” “health,” “exercise,” “food,” “eat,” “weight loss,” and “management;” and the highest eigenvector centrality, followed by “obesity,” “health,” “exercise,” “food,” “management,” “eat,” and “weight loss.”

**Table 3:** Centralities of keywords related to adolescents’ diets from News Network.

Rank	Keyword	Degree centrality	Keyword	Betweenness centrality	Keyword	Closeness centrality	Keyword	Eigenvector centrality
1	increase	0.980	increase	0.055	increase	0.980	increase	0.204
2	obesity	0.959	appearance	0.053	obesity	0.961	obesity	0.203
3	health	0.959	health	0.045	health	0.961	health	0.203
4	exercise	0.939	obesity	0.043	exercise	0.942	exercise	0.201
5	food	0.898	exercise	0.041	food	0.907	food	0.197
6	eat	0.878	food	0.028	eat	0.891	management	0.195
7	weight loss	0.878	follow	0.025	weight loss	0.891	eat	0.195
8	management	0.878	management	0.025	management	0.891	weight loss	0.195
9	follow	0.857	eat	0.024	follow	0.875	follow	0.192
10	effect	0.796	weight loss	0.024	effect	0.831	effect	0.186
11	intake	0.776	treatment	0.019	intake	0.817	fat	0.183
12	fat	0.776	effect	0.014	fat	0.817	intake	0.182
13	treatment	0.776	problem	0.013	treatment	0.817	treatment	0.176
14	problem	0.735	intake	0.013	problem	0.790	problem	0.175
15	weight	0.714	fat	0.012	weight	0.778	needed	0.172
16	needed	0.694	weight	0.011	needed	0.766	weight	0.169
17	appearance	0.673	product	0.007	appearance	0.754	make	0.169
18	make	0.673	use	0.007	make	0.754	use	0.162
19	use	0.653	needed	0.006	use	0.742	appearance	0.155
20	product	0.592	make	0.005	product	0.710	method	0.153
21	method	0.592	education	0.005	method	0.710	life	0.145
22	meal	0.551	method	0.003	meal	0.690	meal	0.145
23	diverse	0.551	vitamin	0.002	diverse	0.690	product	0.144
24	life	0.551	diverse	0.002	life	0.681	diverse	0.144
25	habit	0.510	program	0.002	habit	0.671	habit	0.137
26	program	0.490	activity	0.002	program	0.662	function	0.130
27	vitamin	0.490	meal	0.002	vitamin	0.653	activity	0.129
28	activity	0.490	life	0.002	activity	0.653	calorie	0.128
29	calorie	0.490	calorie	0.001	calorie	0.653	program	0.125
30	function	0.469	habit	0.001	function	0.645	menu	0.124
31	menu	0.469	menu	0.001	menu	0.645	vitamin	0.124
32	education	0.429	function	0.000	education	0.636	stress	0.106
33	protein	0.388	school	0.000	investigation	0.613	prohibition	0.106
34	milk	0.367	milk	0.000	prohibition	0.613	investigation	0.106
35	investigation	0.367	protein	0.000	protein	0.613	protein	0.105
36	prohibition	0.367	prevention	0.000	milk	0.605	education	0.105
37	stress	0.367	consult	0.000	stress	0.605	prevention	0.101
38	prevention	0.347	prohibition	0.000	prevention	0.598	milk	0.099
39	eat nothing	0.327	eat nothing	0.000	school	0.590	eat nothing	0.090
40	school	0.306	take dose	0.000	eat nothing	0.590	side effect	0.085
41	side effect	0.286	investigation	0.000	side effect	0.583	school	0.083
42	take dose	0.245	stress	0.000	consult	0.570	muscle	0.074
43	consult	0.245	advertisement	0.000	take dose	0.563	take dose	0.072
44	muscle	0.245	anorexia	0.000	muscle	0.563	plan	0.067
45	plan	0.224	information	0.000	plan	0.557	consult	0.064
46	anorexia	0.184	side effect	0.000	anorexia	0.551	anorexia	0.054
47	information	0.184	video	0.000	information	0.544	information	0.054
48	advertisement	0.102	muscle	0.000	advertisement	0.527	advertisement	0.030
49	video	0.082	plan	0.000	video	0.516	video	0.023
50	entertainer	0.020	entertainer	0.000	entertainer	0.434	entertainer	0.005

Table 4 shows the network centralities analyzed using the keyword COM for the blogs. As the keyword “weight loss” had the most connections with other keywords, it had the highest degree centrality, followed by “exercise,” “obesity,” “health,” “eat,” “management,” “weight,” and “food”; the highest betweenness centrality, followed by “exercise,” “obesity,” “health,” “eat,” “management,” “weight,” and “follow”; the highest closeness centrality, followed by “exercise,” “obesity,” “health,” “eat,” “management,” “weight,” and “food”; and the highest eigenvector centrality, followed by “obesity,” “health,” “exercise,” “eat,” “management,” “weight,” and “food.”

**Table 4:** Centralities of keywords related to adolescents’ diets from Blog Network.

Rank	Keyword	Degree centrality	Keyword	Betweenness centrality	Keyword	Closeness centrality	Keyword	Eigenvector centrality
1	weight loss	1.000	weight loss	0.075	weight loss	1.000	weight loss	0.210
2	exercise	0.959	exercise	0.064	exercise	0.961	obesity	0.208
3	obesity	0.959	obesity	0.046	obesity	0.961	health	0.206
4	health	0.939	health	0.042	health	0.942	exercise	0.205
5	eat	0.918	eat	0.035	eat	0.925	eat	0.205
6	management	0.898	management	0.028	management	0.907	management	0.204
7	weight	0.878	weight	0.026	weight	0.891	weight	0.201
8	food	0.837	follow	0.019	food	0.860	food	0.197
9	effect	0.796	food	0.018	effect	0.831	effect	0.191
10	intake	0.755	effect	0.015	intake	0.803	intake	0.184
11	method	0.755	function	0.015	method	0.803	method	0.182
12	prescription	0.694	method	0.015	prescription	0.766	prescription	0.171
13	follow	0.694	intake	0.012	follow	0.766	worry	0.168
14	increase	0.653	prescription	0.010	increase	0.742	increase	0.166
15	function	0.653	consult	0.006	function	0.742	follow	0.164
16	worry	0.653	increase	0.006	worry	0.742	needed	0.164
17	needed	0.633	worry	0.006	needed	0.731	function	0.158
18	consult	0.612	use	0.006	consult	0.721	use	0.157
19	fat	0.612	fat	0.006	fat	0.721	appearance	0.156
20	use	0.612	needed	0.005	use	0.721	fat	0.156
21	appearance	0.592	appearance	0.004	appearance	0.710	consult	0.154
22	habit	0.571	habit	0.004	habit	0.700	habit	0.148
23	take dose	0.531	program	0.003	take dose	0.681	meal	0.142
24	meal	0.531	take dose	0.003	meal	0.681	take dose	0.140
25	product	0.510	video	0.003	product	0.671	product	0.137
26	treatment	0.490	diverse	0.003	treatment	0.662	treatment	0.135
27	problem	0.469	meal	0.002	problem	0.653	problem	0.129
28	diverse	0.469	ingredient	0.002	diverse	0.653	diverse	0.126
29	program	0.429	muscle	0.002	program	0.636	ingredient	0.116
30	side effect	0.429	problem	0.002	side effect	0.636	herbal medicine	0.114
31	ingredient	0.429	side effect	0.002	ingredient	0.636	menu	0.114
32	school	0.408	school	0.002	school	0.628	side effect	0.113
33	herbal medicine	0.408	product	0.002	herbal medicine	0.628	program	0.112
34	menu	0.408	calorie	0.001	menu	0.628	make	0.109
35	calorie	0.388	treatment	0.001	calorie	0.620	school	0.108
36	make	0.388	make	0.001	make	0.620	calorie	0.106
37	video	0.367	menu	0.001	video	0.613	physical constitution	0.097
38	physical constitution	0.327	konjac	0.001	physical constitution	0.598	video	0.093
39	activity	0.327	activity	0.001	activity	0.598	life	0.093
40	muscle	0.306	herbal medicine	0.000	muscle	0.590	activity	0.092
41	life	0.306	posture	0.000	life	0.590	stress	0.088
42	konjac	0.286	appetite suppressant	0.000	konjac	0.583	muscle	0.081
43	stress	0.286	friend	0.000	stress	0.583	konjac	0.076
44	appetite suppressant	0.245	advertisement	0.000	appetite suppressant	0.570	appetite suppressant	0.065
45	friend	0.224	stress	0.000	friend	0.563	friend	0.064
46	posture	0.184	inquiry	0.000	posture	0.551	skip a meal	0.047
47	advertisement	0.143	correction	0.000	advertisement	0.538	posture	0.045
48	correction	0.143	life	0.000	correction	0.538	advertisement	0.041
49	skip a meal	0.143	physical constitution	0.000	skip a meal	0.538	correction	0.035
50	inquiry	0.041	skip a meal	0.000	inquiry	0.510	inquiry	0.014

### Semantic network of clusters from CONCOR analysis related to adolescents’ diets

**Fig 2:**
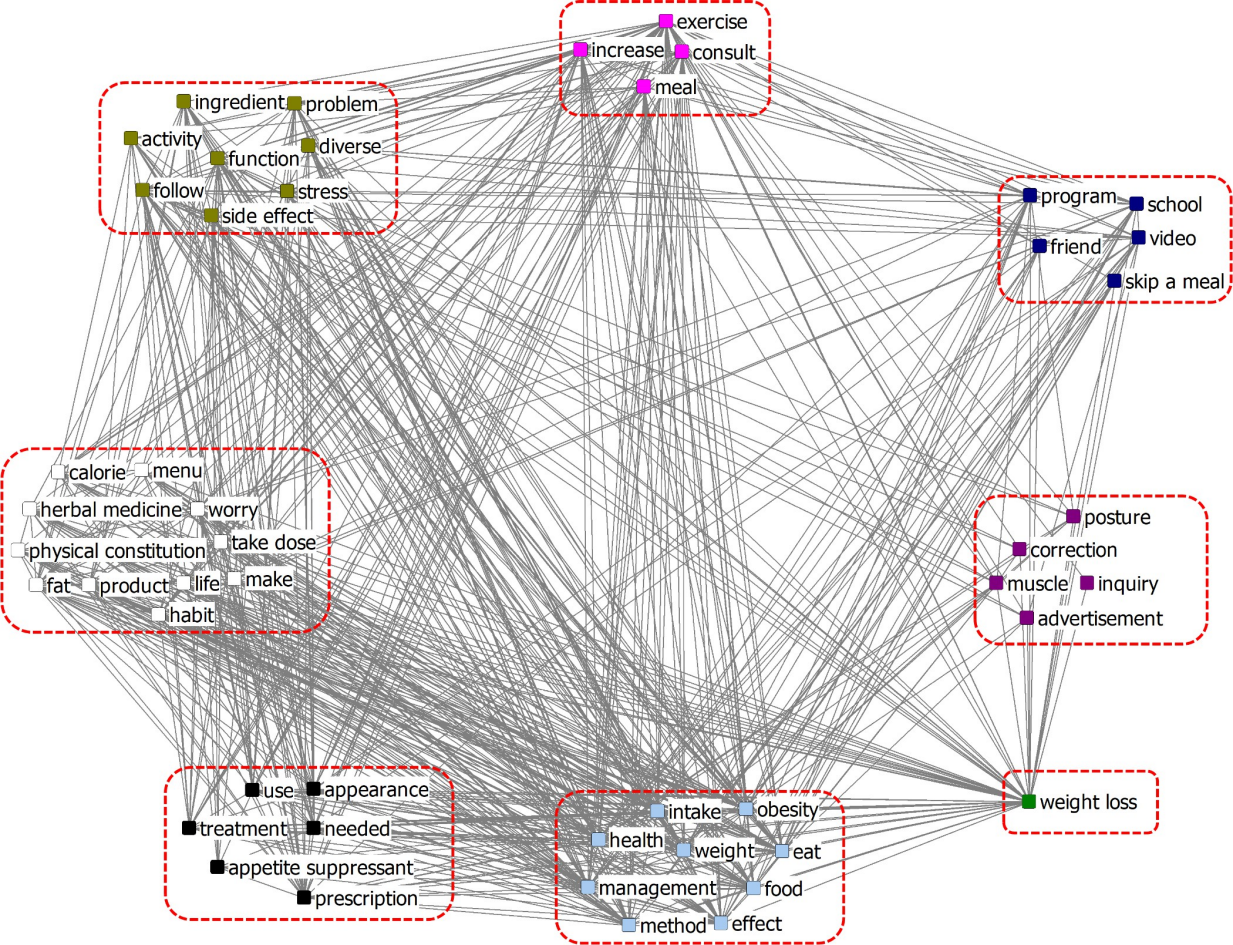
CONCOR analysis of News Network of the adolescents’ diet network.

CONCOR analysis was conducted on cluster words based on their structural equivalence relationship by analyzing Pearson’s correlation in COM. Fig 2 shows the results of the CONCOR analysis of adolescents’ diet network constructed from online news, called News Network, and the eight clusters that were identified. We represented the cluster consisting of *word1*, *word2*, … in [*word1*, *word2*, …]. The cluster [menu, vitamin, calorie, meal] could be seen as a collection of words related to “*how to eat*.” The cluster [weight loss, eat, obesity, food, follow] could be interpreted as *“eating foods that do not contribute to obesity is followed by weight loss*.” The cluster [problem, need, make, fat, weight, intake, effect] could be regarded as *“the effects of the intake of fats that makes it a weight problem*.” The cluster [plan, take dose, muscle, eat nothing, milk, protein, anorexia, prevention, side effect, prohibition, stress] consisted of words related to “*side effects and their prevention*.” The cluster [management, exercise, health, increase] was related to “*the importance of increasing health management and exercise*.” The cluster [habit, function, activity, use, investigation, life, appearance, method, diverse] could be interpreted as “*things to be done by activating the body*.” The cluster [video, program, education, information, school, product, consult, treatment] emphasized “*education and information*.” The cluster [entertainer, advertisement] could be seen as “*influencing factors*.”

**Fig 3:**
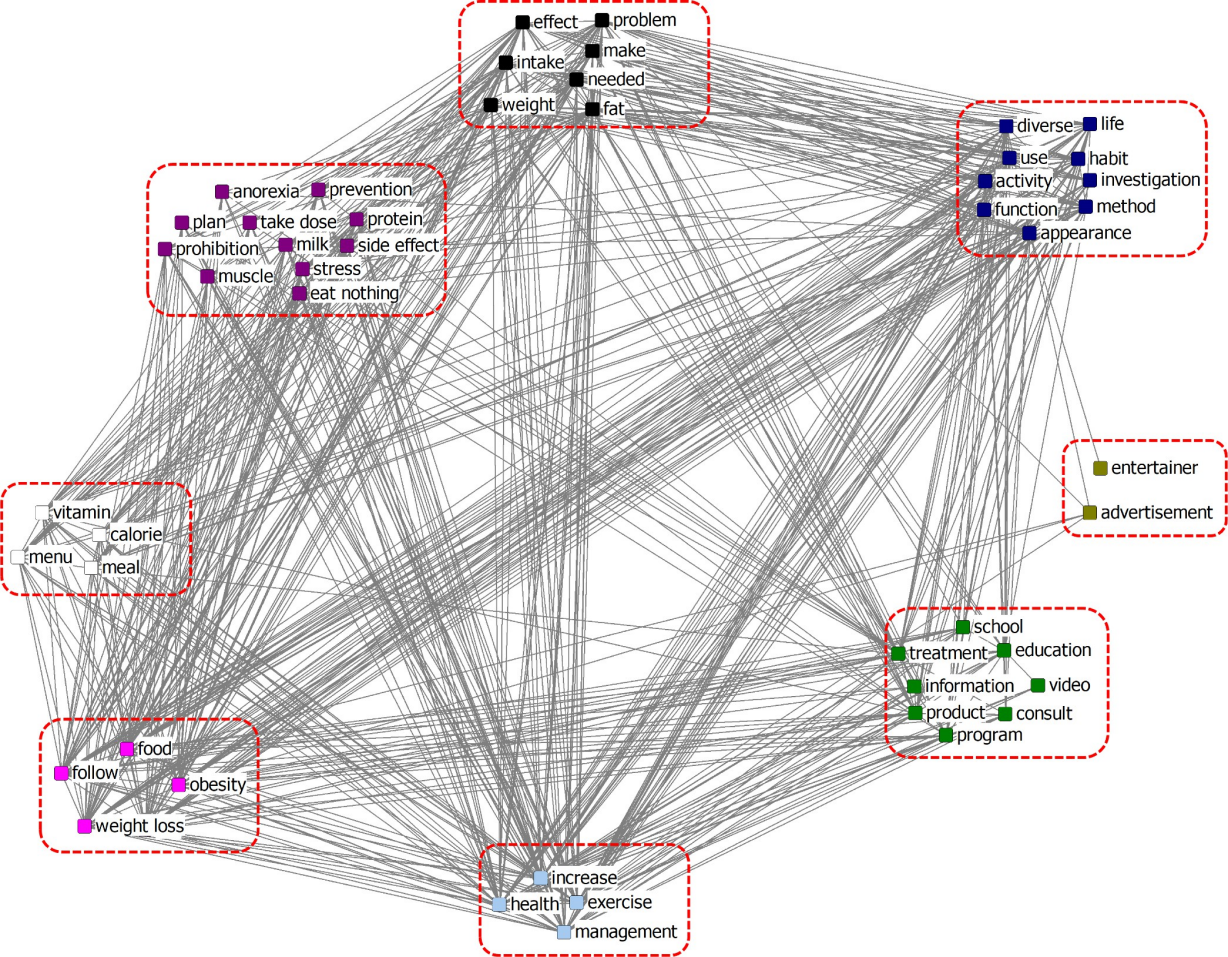
CONCOR analysis of Blog Network of the adolescents’ diet network.

Fig 3 shows the results of the CONCOR analysis of the adolescents’ diet network constructed from blogs, called Blog Network, and the eight clusters that were identified. The cluster [calorie, worry, physical constitution, life, take dose herbal medicine, habit, menu, make, konjac, product] could be seen as words related to “*eating habits*.” The cluster [treatment, appetite suppressant, use, needed, appearance, prescription] referred to “*methods*, *except food and exercise*.” The cluster [meal, consult, exercise, increase] could be interpreted as “*it is desirable to increase consultations on exercise and meals*.” The cluster [function, diverse, problem, side effect, follow, activity, ingredient, stress] was regarded as “*problems that could occur from a diet*.” The cluster [weight, health, management, eat, intake, food, obesity, effect, method] could be interpreted as the “*importance of a diet*.” The cluster [program, school, video, friend, skip a meal] could be seen as “*social factors that influence skipping a meal*” in adolescents’ diets. The cluster [muscle, inquiry, posture, advertisement, correction] was related to “*body shape*.” The cluster [weight loss] suggested that “*weight loss*” was important.

## Discussion

This study was conducted to provide basic data for establishing a strategy for preventing adolescent obesity, which is increasing yearly, and establish desirable weight control strategies by analyzing online data on the diet behaviors of adolescents using text-mining techniques.

Among the words extracted by text mining on adolescents’ diets, the top five words with high frequency were “obesity,” “health,” “exercise,” eat,” and “increase” in online news, and “exercise,” “eat,” “weight loss,” “obesity,” and “health” in blogs. This result was consistent with those of a study, in which “exercise” and “health” were the keywords with the highest frequency in the 2016 diet status analysis through big data by selecting Naver, the most used portal in Korea, as an analysis target [31]. In a previous study, the word “menu” was included in the top three, whereas in this study, the word “menu” was ranked relatively low—38^th^ and 34^th^ for online news and blogs, respectively. Although the previous study [31] had no age restrictions, and this study was limited to adolescents, both studies have shown high frequencies of “exercise” and “health.” These results suggest that diet is beneficial for health, regardless of age, and in relation to diet, exercise is the most important factor.

What stood out in the centrality analysis of online news was that the betweenness centrality of “appearance” was particularly higher than that of the other centralities. Thus, it can be considered that appearance acts as a bridge connecting others. For example, in the centrality analysis of keywords extracted from online news, the significance of “appearance” in adolescents’ diet behaviors, such as considering themselves obese after seeing an entertainer’s appearance, or choosing diet products after seeing advertisements, was confirmed. Adolescence is a period of rapid physical growth and social development, when interest in one’s appearance increases. Adolescents’ values and attitudes toward their appearance are easily influenced by the mass media or their peer groups. Additionally, as this study’s results have been supported by studies stating that even non-obese adolescents are highly preoccupied with their appearance, such as erroneously recognizing their body type as being obese, it shows that the betweenness centrality of “appearance” is particularly high in centrality analysis [24, 32]. Disordered weight control behaviors should be considered when developing education programs to establish desirable weight control, given their prevalence among Korean adolescents [33], and their association with stress and depressive symptoms [24, 34].

In this study, the issues identified from the CONCOR cluster analysis of online news and blogs were somewhat different. Based on the results of the CONCOR cluster analysis of keywords extracted from online news, the following can be inferred regarding intervention in adolescents’ diet behaviors. First, during diet interventions, emphasizing education on side effects and how to prevent them is necessary. Second, entertainers and advertisements can affect adolescents’ diets, so this point should be reflected in diet-related education. Third, referring to online news rather than blogs is better because online news has more content on diet-related education and information.

Obesity treatment drugs have problems of side effects and abuse, and especially since a large-scale clinical study has not yet been conducted for adolescents, more attention is required. In contrast, the spread of a distorted sense of beauty in favor of an overly skinny body encourages the indiscriminate use of anti-obesity drugs; hence, safety issues are constantly being raised related to the overuse, dependence, and misuse of psychotropic appetite suppressants [35, 36]. Therefore, it supports this study’s results, showing a significant interest in side effects and their prevention, following the use of therapeutics for weight loss. Diet inspiration-related information or slender models seen in the media affect individuals’ perceptions of their body image, which also affects their self-attitudes, such as body dissatisfaction [37, 38]. This is consistent with this study’s results, in which appearance had the second highest value in the betweenness centrality analysis of online news, and the results of the CONCOR analysis showed that a cluster consisting of entertainers and advertisements could influence adolescents’ diets. Another result confirmed in the CONCOR analysis of online news was that many content items were related to diet-related education and information. This is supported by the statement that online newspapers lend themselves to be used as a “research medium” for more information on issues that one is already interested in [39].

Based on the results of the CONCOR analysis of keywords extracted from blogs, the following can be inferred regarding the intervention in adolescents’ diet behaviors. First, it is necessary to emphasize the importance of food intake and diet for weight control. Second, it has been confirmed that adolescents have so much interest in body shape that this point will be reflected in the intervention. Third, since there is a lot of information about weight loss in blogs, it is necessary to reflect on information and education with reference to them.

Comparing the results of the CONCOR analysis of online news and of blogs, online news contained more education and information, such as how to eat, non-obese food, and the side effects of diet (weight control), whereas blogs contained more content on intake, body shape, and weight loss. This suggests that the differences in authors’ subjective thoughts and direct experiences are used as the main basis for blogs, whereas online news is focused on delivering objective information and explanations, based on the values of fairness and responsibility [40].

Diet and food-related content on social media may influence people’s diets and weight-loss behaviors. Visual cues, such as images or videos of food, increase the likelihood of eating and gaining weight [41]. Moreover, research has shown increased marketing potential for unhealthy foods and beverages through social media [42]. This is consistent with the results of the CONCOR analysis of keywords extracted from blogs, showing that education on the importance of intake and diet should be emphasized. Adolescents are interested in weight control and prefer a skinny body; and even if their weight falls within the standard range or below, they still want to lose weight and follow a diet [32]. This supports the results of the CONCOR analysis of the keywords extracted from blogs in this study. Adolescents’ subjective perceptions of being underweight and overweight were positively associated with problematic Internet use. Considering this, careful attention needs to be paid to adolescents’ inappropriate weight control behaviors [43].

Although adolescence is a period in which physical and physiological growth along with development must be sufficiently achieved, excessive expectations for a slim body are highly likely to cause physical and psychological problems, such as damaging health and lowering self-esteem. Therefore, to prevent problems caused by extreme and excessive weight loss, it is necessary to provide reasonable monitoring standards for the media mainly used by adolescents, such as TV and the Internet, as well as education to critically select information and properly accept it. Furthermore, correct and educational information should be provided, so that adolescents can have more positive self-perceptions and personal satisfaction about their physical appearance, and thereby establish a desirable self-identity.

## Conclusions

Although information related to adolescents’ diets is widely available on the Internet, we collected data from Naver News, Naver blogs, and Daum blogs to obtain better crawling results, given that Google search results on this topic are mainly news and blogs. This study was limited by its search terms. Data were collected using the search term “adolescents’ diets,” along with similar and related words. The collected data may depend on the range of the similar words that were selected. In this study, data were collected only in the Korean language from Korean portal sites. Although information on adolescents’ diets are available from websites worldwide, the data were collected in a single language to guarantee consistency with keyword selection. Despite these limitations, this study’s outcomes were significant. As it analyzed data extracted from online news and blogs, its results will serve as a basis for intervention strategies for weight management, reflecting the perspectives of adolescents, who have a high rate of weight loss attempts, and spend a lot of time on smartphones. Its results can also be used as basic data to help establish and provide correct information to adolescents for establishing desirable weight control in the future and helping them to grow into healthy adults.
